# Psychological occupational syndromes in UK emergency medicine doctors: a scoping review

**DOI:** 10.1093/occmed/kqag080

**Published:** 2026-09-28

**Authors:** E Beck, J Daniels

**Affiliations:** Department of Psychology, University of Bath, Claverton Down BA2 7AY, UK; Department of Psychology, University of Bath, Claverton Down BA2 7AY, UK

## Abstract

**Background:**

Doctors working in UK emergency departments face high workloads, stretched resources and threats to career sustainability. Prolonged stress in the context of high workloads is associated with the development of psychological occupational syndromes such as burnout and compassion fatigue.

**Aims:**

This scoping review aimed to map the existing literature available to provide an understanding of the psychological occupational syndromes experienced by UK doctors working in emergency medicine.

**Methods:**

A systematic search was conducted using PubMed, Embase, APA Psycnet and CINAHL. Reference lists and supplementary searches were also conducted.

**Results:**

Seventeen papers were eligible for inclusion in this review. Factors highlighted as relevant to the experience of psychological occupational syndromes included organizational barriers, team functioning, personal and demographic factors, and the nature of the emergency department environment. No research identified in this review looked at whether issues of equality, diversity or inclusion (EDI) were related to the experience of psychological occupational syndromes.

**Conclusions:**

There are similarities across psychological occupational syndromes in terms of how they are experienced by emergency medicine doctors and their consequences; however, there is a lack of research determining causality over time, looking at multiple psychological occupational syndromes together and considering EDI factors, which limits the ability to draw firm conclusions. The findings of this review align with existing research and identify gaps in the evidence base that should be addressed with future research.

## Teaser

This research reviews 17 papers that explored different conditions of occupational stress in UK emergency medicine (EM) doctors, including burnout and compassion fatigue. The findings align with global research, highlighting issues including organisational barriers, team functioning and personal and demographic characteristics as key to the experience of psychological occupational syndromes in UK EM doctors. Multi-level solutions are required to tackle this, as well as further research to address critical knowledge gaps.

Key learning points **What is already known about this subject:**

- Doctors working in emergency medicine (EM) are required to provide lifesaving care in a fast-paced environment, whilst trying to navigate a lack of resources and inadequate staffing levels.
- Research has highlighted that EM doctors are more at risk of experiencing burnout compared with other specialties or professions.

**What this study adds:**

- This scoping review adopted a broader approach to occupational stress in EM doctors to assess what is already known on this topic and to synthesize findings across psychological occupational syndromes and varying methodological designs.
- There are several factors that appear to be relevant across the psychological occupational syndromes experienced by EM doctors, which can be categorized into issues related to organizational barriers, personal characteristics, team functioning and the inherent nature of the emergency department environment.
- This review has identified crucial gaps in the literature that can inform future research and enable a more comprehensive understanding of the psychological occupational syndromes experienced by EM doctors.

**What impact this may have on practice or policy:**

- Further research is needed to better understand psychological occupational syndromes specifically in this group, to ensure that practices and policies meet the needs of all doctors working in EM.
- To effectively address the psychological occupational syndromes experienced by EM doctors it is likely that changes are required at multi-system levels, but system changes may have the biggest impact.
- Workplace culture changes may be helpful in reducing some of the stigma around experiencing syndromes such as burnout. This could include EM leaders prioritizing team morale and psychological wellbeing, to help build team cohesiveness and increase EM doctors’ feelings of being valued for the work they do.

## Introduction

UK Government frameworks outline the need for research-informed policies that improve National Health Service (NHS) culture, staff wellbeing and ensure the delivery of optimal patient care [1,2]. Whilst these issues are prevalent across the NHS, priority should be given to those areas most in need. Emergency departments (EDs) are 24/7, high-pressure, unpredictable clinical environments critical to the health and wellbeing of the nation. The stakes in Emergency Medicine (EM) are high, yet doctors are required to provide urgent care to acutely unwell patients with compromised staffing levels and resources, within the context of unsustainable working practices [3]. Unsurprisingly, EM doctors are at high risk of poor health and wellbeing [4–6] and report a higher need for recovery compared to any other reported profession or population [7].

An occupational phenomenon consistently linked to workforce sustainability in the EM workforce is burnout [4,8], characterized by the experience of emotional exhaustion, de-personalization and perceptions of reduced professional accomplishment [9,10]. There is a pressing need to address burnout across the NHS [4,6,9] and EM doctors are among those reporting the highest levels [3,4,6,11]. Burnout compromises concentration and executive functioning ability [12], which is vital to decision-making; it is also associated with negative thinking and cognitive biases [13], which can lead to feelings of reduced personal accomplishment and self-efficacy. Consequences of burnout are multifarious and can lead to medical errors, attrition and financial implications [4,6,9,14].

Other occupational syndromes, including compassion fatigue, alienation, moral injury and embitterment, result in similar consequences [9] and present with overlapping sub-characteristics of burnout [15–19]. However, moral injury is distinct in that the experience of distress is in direct response to witnessing or participating in an event that violates one’s moral beliefs [20]. Relatedly, embitterment occurs when professionals feel that they have experienced unfair treatment from their employer [21]. The overlap in the clinical presentation of several of these syndromes indicates the appropriateness of considering these conditions together as ‘psychological occupational syndromes’. As we seek solutions to the strained workforce and seek evidence to underpin key policies from which we can develop targeted key interventions, we must first understand the impact and relevance of these psychological occupational syndromes and their contribution to EM.

When considering other relevant factors related to psychological occupational syndromes, attention is owed to issues of equality, diversity and inclusion (EDI). The COVID-19 pandemic highlighted health inequalities experienced by healthcare staff from diverse backgrounds and the impact on physical and emotional wellbeing [22]. Research has reported that NHS staff from minority backgrounds are more likely to experience poorer mental health [23], and it may be that EDI intersects with, and exacerbates difficulties such as burnout and compassion fatigue. Assessing the current state of the evidence will provide a further understanding of psychological occupational syndromes and their impact. Previous similar reviews have focused on a range of healthcare professionals [16] or doctors working in EDs globally [8,24–26]. Research has highlighted organizational factors such as heavy workload, feeling undervalued and workplace conflict as key factors contributing to burnout [25], and that there is a high probability between the development of burnout and patient safety issues [26].

Another review found that working in the emergency department was indicative of higher levels of compassion fatigue and burnout in nurses, whilst also acknowledging that a better understanding of compassion fatigue and related concepts is required for successful prevention and intervention [16]. Narrowing the focus to a specific professional group, whilst definitions and understanding of these concepts are so broad and variable, could act as a good starting point for increasing clarity.

Retention of EM doctors is a global issue, with burnout playing an important role [8]. If EM doctors are leaving their careers earlier due to issues such as burnout, work-related stress and resulting reduced job satisfaction, EDs are at risk of having less senior and experienced staff, potentially reducing the delivery of high-quality care for patients [8]. Furthermore, with higher levels of career attrition comes higher turnover costs for healthcare systems that are already financially stretched.

Additionally, a meta-analysis has indicated that the global prevalence of burnout among EM healthcare workers is high [27]. Studies that looked only at EM physicians reported higher burnout prevalence compared to studies which involved multiple EM healthcare professions [27], indicating the importance of understanding the needs of different professional groups. Further differences between countries in terms of burnout rates were highlighted [27], demonstrating the importance of understanding these issues both globally and applied to individual countries. Moreover, only one research paper in this meta-analysis was UK-based, highlighting a gap in our UK/NHS-specific understanding of these issues. This is important as there is a pronounced need for UK-wide standards when it comes to supporting NHS doctors [6] and providing specific interventions and policies tailored to the needs of this neglected group.

In consideration of these issues, this scoping review aims to use a systematic approach to answer the following questions: firstly, *‘What do we know about the psychological occupational syndromes experienced by UK EM doctors and how can we use this knowledge to inform future research and policy guidelines?’*; and secondly,* ‘Are issues of equality, diversity and inclusion relevant when considering the psychological occupational syndromes experienced by UK EM doctors?*

## Methods

A scoping review was deemed appropriate due to the exploratory nature of the research questions and the main aim of exploring the existing literature on ‘psychological occupational syndromes’ as applied to UK EM doctors. Due to the differing definitions and concepts used in the wider literature, a scoping review would enable us to map the current evidence base on this topic for a specific group and aid the identification of critical knowledge gaps that should be addressed with novel research. Further, a scoping review allows for a comprehensive approach to evidence sources [28]; this review included quantitative, qualitative and mixed-methods research studies, opinion pieces and grey literature. Grey literature is information that is produced outside of traditional publishing channels or peer review, including resources such as blog posts. This review was conducted in line with the PRISMA-ScR checklist (Preferred Reporting Items for Systematic Reviews and Meta-Analyses extension for Scoping Reviews) [29] and the Joanna Briggs Institute (JBI) framework [30,31]. The protocol was pre-registered and is available on the Open Science Framework registry [32]. Eligibility criteria are presented in Table 1. Papers were excluded if they did not meet the eligibility criteria, were review articles or were research studies designed to investigate the effectiveness of different interventions for psychological occupational syndromes.

**Table 1:** Eligibility criteria in line with the PCC (population, concept, context) framework [30]

Population/participants	UK based doctors working in emergency medicine
Concept	EM doctors’ experiences of ‘psychological occupational syndromes’ defined as concepts related to poorer wellbeing of doctors in emergency medicine as a result of their job: Burnout Compassion fatigue Moral injury Psychological distress/work related stress Need for recovery Emotional exhaustion Depersonalization/cynicism Reduced sense of accomplishment and professional efficacy Embitterment Alienation Moral distress
Context	Environment of UK emergency departments. Issues related to equality, diversity and inclusion were also explored, if related to UK EM doctors. The context of pre, during and post COVID-19 lockdown restrictions was considered, to explore whether the description of ‘psychological occupational syndromes’ changed across the years.

Systematic searches of the literature were conducted on 20 November 2024 utilizing PubMed, Embase, APA Psycnet and CINAHL. A Google Advanced supplementary search was used to identify grey literature, linking key search terms to relevant domains including the BMA (British Medical Association) and the RCEM (Royal College of Emergency Medicine). This was done in line with JBI guidelines for searching for grey literature and reviewing the first 5-10 pages of the search [30] and with a previously published scoping review [8]. The same inclusion and exclusion criteria were applied when searching for grey literature. The full search strategy for PubMed is reported in Table 2 (all search strategies are included in the Supplementary Material, available as Supplementary material at *Occupational Medicine* Online). Reference lists of eligible papers were screened to identify additional sources. No date limits were applied to the searches due to the purpose of assessing all available evidence. English language limits were applied as the review focused on UK EM doctors.

**Table 2:** Full search strategy for PubMed (including Medline)

Population	doctor*[tiab] OR “Physicians”[Mesh] OR physician*[tiab] OR registrar*[tiab] OR trainee*[tiab] OR consultant*[tiab]
AND	
Concept	“Burnout, Psychological”[Mesh] OR “Burnout, Professional”[Mesh] OR depersonalization[Mesh] OR “compassion fatigue”[Mesh] OR “occupational stress”[Mesh] OR burnout[tiab] OR “compassion fatigue”[tiab] OR “occupational stress”[tiab] OR “emotional exhaustion” [tiab] OR cynic*[tiab] OR depersonalisation[tiab] OR depersonalization[tiab] OR “reduced personal accomplishment” [tiab] OR “professional efficacy” [tiab] OR embitterment[tiab] OR alienation[tiab] OR “moral injur*” [tiab] OR “empathy fatigue” [tiab] OR estrangement [tiab] OR “job stress”[tiab] OR “work stress”[tiab] OR “job burnout”[tiab] OR “moral distress”[tiab]
AND	
Context	“emergency medicine”[Mesh] OR “emergency service, hospital”[Mesh] OR “trauma centers”[Mesh] OR “emergency treatment”[Mesh] OR “emergency medical services”[Mesh] OR “emergency medical service*”[tiab] OR “emergency medic*”[tiab] OR “emergency service*”[tiab] OR “trauma cent*”[tiab] OR “emergency department*”[tiab] OR “emergency treatment*”[tiab] OR “emergency care”[tiab] OR “accident and emergency”[tiab] OR “A&E”[tiab] OR “emergency room*”[tiab] OR “emergency ward*”[tiab] OR “emergency health service*”[tiab] OR “hospital emergency service*”[tiab]
*Data extraction*	
Items for extraction	Author(s) Publication year Type of paper Research question/aims Area of UK Type of emergency department Participant demographics Methodology Measures Data collection methods Outcome Time points collected Data analysis approach Key findings Key conclusions Any other relevant information

All search results were uploaded into Endnote 20 and duplicates were removed before importing into Covidence. The full screening process was carried out independently by EB (lead researcher) and BG (research assistant), with conflicts being resolved by EB and JD (supervisor). All titles and abstracts were screened by EB and BG against the inclusion criteria. Full texts were then screened against the inclusion criteria and those that were not eligible were removed. Cohen’s Kappa was 0.96 for full-text screening, indicating almost perfect agreement between reviewers.

The data extraction form (available in the Supplementary Material, at *Occupational Medicine* Online) was piloted by EB and BG to check agreement. Data extraction was carried out independently for all papers by EB and BG. Consensus was assessed and agreed by EB. The data extraction tool was adapted from the JBI data extraction instrument [29] and the types of data extracted are included in Table 2.

Due to the breadth of psychological occupational syndromes explored in this review and the inclusion of different evidence types, adopting an inductive approach to conducting the narrative synthesis permitted findings, concepts and themes to be derived, synthesized and clarified across different methodological approaches and diverse data. However, EB held pre-existing knowledge which may have had some influence on the analysis. Narrative synthesis was conducted in line with published guidance and adapted for the specific review questions [33–35]. A preliminary synthesis was developed which allowed descriptions of each paper to be devised, and for identification of possible themes or concepts related to the experience of psychological occupational syndromes. This included using groupings, tabulation and conventional content analysis. EB familiarized herself with the papers and then counted the frequency with which particular themes or concepts were present. Next, EB explored and identified factors that could clarify similarities or differences in findings, such as those related to methodology. Data synthesis was conducted as an iterative process, with the initial focus placed on the research studies included in the review to develop understanding and draw conclusions based on empirical evidence. The expert opinion pieces (including grey literature) were included in the analysis to contribute to the narrative and allow for more depth and nuance across the themes and concepts. Additionally, although not required for scoping reviews, quality appraisals were made using the JBI critical appraisal checklists [36–38] by EB and BG. No cut off was applied to ensure that this review included all available evidence.

## Results

Initial database searches identified 2730 sources and supplementary searches identified seven (see Figure 1 for the flow chart). Following removal of duplicates, 2083 titles and abstracts were screened. Of those, 1832 were removed due to not meeting the eligibility criteria. Full text screening was undertaken on 249 sources, which identified 15 eligible sources. Reference list searches identified a further two sources, resulting in 17 for data extraction. Of the final 17, three were conference abstracts. Requests were sent to the study authors for the full papers. However, these were not received. Table 3 (available as Supplementary data at *Occupational Medicine* Online) summarizes each paper.

**Figure 1:**
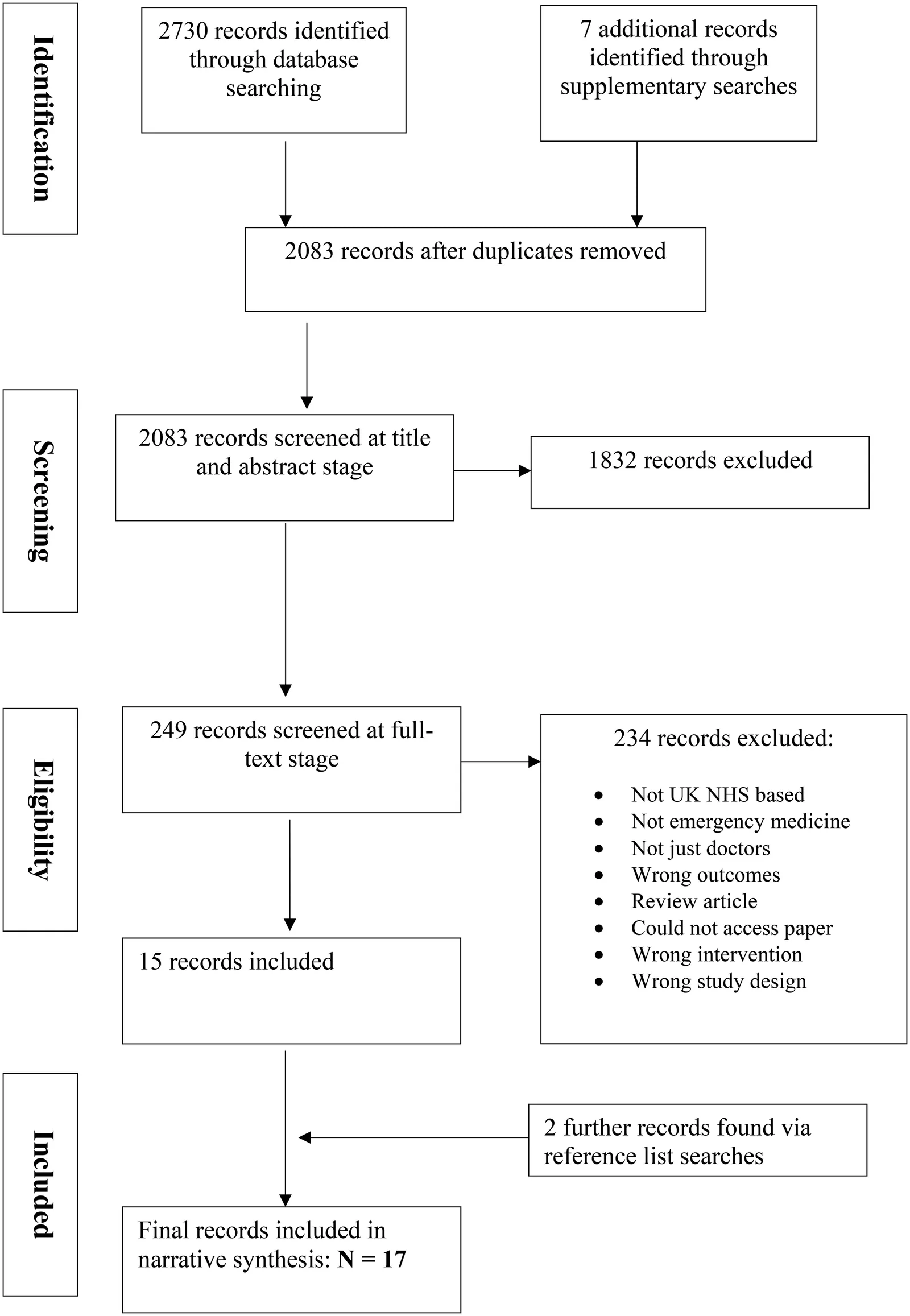
Flowchart adapted from PRISMA-ScR [29].

Of the seven quantitative studies [39–45], three provided limited demographic information [40–42], limiting the studies’ ability to consider potential confounding variables. Two included sources were conference abstracts [44,45], making it difficult to complete a full quality appraisal. There is a risk of social desirability bias due to the reliance of all studies on self-report.

Qualitative papers (*N *= 3) included in this review [46–48] appear to be of good quality, according to the JBI appraisal tools. Congruence between perspectives, methodology, analysis and interpretation was evident for the studies [46–48]. However, one was presented as a conference abstract so information was limited [48]. Participants’ voices were adequately represented throughout all three papers, but only one acknowledges the possible influence of the researcher (an EM doctor) on participants [47].

The two mixed-methods papers gave some description of demographic information, but ethnicity was not reported by either [49,50]. One of these used validated measures [49] and the other developed the survey for the study, based on a published literature review [50]. Sampling bias is a possibility, particularly considering the 52% response rate in one paper [49]. Considering the qualitative aspects of these papers, one conducted interviews and demonstrated congruity between objectives and methods [49], and the other used thematic analysis of open-ended survey questions [50].

Five expert opinion pieces were included in this review. All experts have standing in the field of EM [51–55]. Only two were published in peer-reviewed journals [54,55] with three being grey literature [51–53]. The three pieces of grey literature came from the RCEM website, and an online blog platform run by specialists in emergency medicine and critical care.

Papers were published between 1993 and 2024. Of the 17 papers, seven were quantitative survey designs [39–45], three were interview studies [46–48], two were mixed-methods [49,50] and five were expert opinion pieces [45,51–55]. Three of the expert opinion pieces were grey literature [51–53]. Further information is reported in Table 3 (available as Supplementary data at *Occupational Medicine* Online).

Outcomes and methods of measurement varied across the research studies (*N *= 12). Main study outcomes included psychological distress or emotional disruption (*n *= 5)[39,40,43,46,47], work-related stress (*n *= 4) [40–42], burnout (*n *= 1) [44], compassion satisfaction (pleasure from helping others) and compassion fatigue (*n *= 2) [49], clinical confidence (*n *= 1) [40], medical information overload (*n *= 1) [50], need for recovery (*n *= 1) [45] and work satisfaction (*n *= 2)[41,42]. Self-report questionnaires were used in 75% of the research studies (*n *= 9) [39–45,49,50], and interviews were used in 33% (*n *= 4) [46–49]. Table 3 (available as Supplementary data at *Occupational Medicine* Online) reports the specific outcomes and measures for each paper.

Of the 17 included papers, 12 were research studies and five were opinion pieces. For the 12 research studies, sample sizes ranged from 10 to 681 (median = 186). Six of the studies recruited participants nationally (50%) [39,41,42,44,45,49] and the others recruited from Northern England [47,50], Edinburgh [48], North London [43], the South Thames Region [40] and South West England [46].

Of the 12 research studies, six investigated junior doctors (50%) [40,41,43–45,48], four investigated consultants (most senior hospital-based surgeon or physician) and senior registrars (doctors undertaking specialty training prior to consultant level) [39,42,46,49], and two included doctors of any level [47,50]. Age of participants was reported in seven of the 12 research studies [39,40,42,43,46,49,50]. Mean age, as reported by four studies, ranged from 26.5 to 43.7 [40,42,43,46]. One study reported a median age of 45, with a range of 31–67 [39] and two reported the percentage of participants in each age category, which ranged from 30–60+ [49] and 20–59 [50]. Of the four studies that reported hours worked per week, between 67 and 100% of participants worked full-time [39,41,46,49]. Marital status was reported in four studies, with the majority of participants stating that they were married or cohabiting (> 83%) [39,42,46,49].

To collate findings in relation to the research questions, a narrative synthesis was conducted for the included sources (*N *= 17).

The reported rates of psychological occupational syndromes varied across the 12 research studies in this review, but no papers considered moral injury, alienation or embitterment. One study reported high levels of burnout in doctors at all stages of specialist training [44] and over 50% of junior doctors met the threshold for psychological distress in another study [43]. The former consisted of a sample of 432 nationally recruited trainees, compared with the latter which included 37 junior doctors from one training session, suggesting that the findings are unlikely to be nationally representative. One study reported that 44% of consultants met the threshold for psychological distress [39]. In contrast, another study did not find particularly high levels of psychological distress in junior doctors [40]. However, the authors developed their own questionnaire and did not offer a statistic for internal consistency [40], which may limit the reliability of their findings. Two studies that assessed work-related strain did not find high levels of work-related stress in junior doctors, senior registrars or consultants [41,42]. One study investigating compassion satisfaction and fatigue in consultants reported average levels of compassion satisfaction and burnout, and low levels of secondary traumatic stress [49]. It is noteworthy that not all EM doctors within the included studies reported high levels of psychological occupational syndromes. This may be due to factors that help maintain improved wellbeing, including adaptive coping strategies, higher clinical confidence, perceived autonomy in the role and a supportive team environment [39–43,46–51,53]. The inconsistent prevalence could also highlight the difficulty in directly comparing results that are taken from different sample sizes and varying methods of measurements. All papers are at risk of sampling bias and studies solely focusing on consultants [39,42,46,49] will not include those who left the career whilst still working as a junior doctor, biasing the conclusions drawn. Considering the included studies, the prevalence of psychological occupational syndromes in EM doctors is estimated to be up to 50%. However, due to the differing sample sizes, levels of seniority and methodology, the actual prevalence is unknown. The factors purported to be relevant to psychological occupational syndromes across all included papers can be categorized into five themes (Table 4).

**Table 4:** Factors related to the experience of occupational syndromes.

Themes and subthemes	Number of sources
**Theme:** Organizational barriers to effective working. **Subthemes:** A lack of financial, practical and personnel resources Unsustainable demands including level of emails, intensity of workload and time pressure and time pressure Lack of clarity around tasks, roles and processes Reduced control and autonomy, including self-rostering	*N *= 7 [39,40,46,49,51,52,55] *N *= 10 [39,40,46,48–52,54,55] *N *= 3 [41,42,48] *N *= 6 [45,46,48–50,55]
**Theme:** Effectiveness of team functioning **Subthemes:** Team cohesiveness/work group functioning and communication difficulties Feeling undervalued, lack of recognition	*N *= 9 [39–42,46–49,51] *N *= 4 [39,46,49,51]
**Theme:** Personal factors **Subthemes:** Coping styles, reluctance to seek help Confidence in own competence	*N *= 6 [43,46,47,49,51,53] *N *= 4 [39,47,48,50]
**Theme:** Demographic factors	*N *= 7 [39,42,44,49,50,52,54]
**Theme:** Nature of the ED environment **Subthemes:** Clinical events and complex presentations 24/7 culture Burden of responsibility, including fear of medical errors and litigation, and leadership	*N *= 2 [39,47] *N *= 4 [46,49–51] *N *= 6 [46,47,49,53–55]

Ten papers highlighted organizational barriers to effective working [39,40,46,48–52,54,55]. Only one paper explicitly reported on personnel levels (ratio of staff to patients) [41] and two explicitly reported on patient attendances per year [41,42] which may highlight the differing ways in which unsustainable demands and lack of resources are referred to within the literature. Six papers referred to the experience of reduced control and autonomy [45,46,48–50,55], but only one considered the difference between those who self-rostered and those who did not, which seems a pertinent factor in the experience of control over one’s work schedule [45]. However, this study did not consider other factors related to staff wellbeing, which may limit the strength of the conclusions drawn [45].

In terms of team functioning, interview participants with higher compassion fatigue were more likely to reflect on negative work relationships with colleagues, and those with higher compassion satisfaction were more likely to reflect on positive work relationships [49].

In two papers, some doctors described having to constantly give more of themselves each day, and how feeling unappreciated by others can threaten their professional identity and reduce job satisfaction [46,51]. Conversely, in one study, despite a lack of recognition being a significant predictor of higher distress scores on the GHQ, 65% of participants reported high satisfaction with the specialty [39].

Adaptive coping strategies such as physical activity were reported to be helpful in coping with the challenges of emergency medicine across four papers [43,46,49,53]. Whilst consultants with higher levels of compassion satisfaction in one study reported the use of adaptive strategies [49], and maladaptive strategies such as venting were significantly related to increased anxiety and depression in another study [43], it is difficult to determine the direction of the relationship between coping strategies and psychological occupational syndromes due to the cross-sectional nature of the studies. Reduced confidence and pressure to impress senior colleagues were related to higher psychological distress [40], handover-related stress [48] and negative effects of information overload [48], reiterating the importance of a supportive team environment, particularly for more junior doctors.

Considering demographics, the experience of psychological occupational syndromes in more junior doctors is concerning [44,52]; in one study, younger doctors reported that the experience of information overload had a bigger impact on their social activities, decision-making and reliance on clinical guidelines, compared to older colleagues [50]. One study included age, gender and years since qualifying in its analysis, although these were separate correlations rather than a regression, which would have identified the amount of variance explained by each variable [39]. One discussion paper emphasized the importance of considering appropriate expectations based on the length of time a doctor has been at a particular grade [54]. This is highlighted by the findings of one study, which reported a non-linear relationship between years as a consultant and compassion satisfaction, where compassion satisfaction reduced over the first 10 years and increased after 20 [49].

Complex presentations and emotionally disruptive events are inherent to EM but may result in psychological distress, as measured both quantitatively and qualitatively [40,47], although not all doctors respond to events in the same way [47]. Some EM doctors may report the burden of responsibility and fear of medical errors or litigation as contributing factors to their work-related stress [46,47,49,53,55] and experience an increase in their own risk aversion as a result [46]. One discussion piece considered how working in the ED may be a double-edged sword, as it is often the nature of the work that attracts doctors to the job but that also leads to burnout, indicating the need for effective leadership and organizational support [54].

Concerns regarding impaired decision-making and reduced quality of patient care were reported across nine papers [39,43,44,46,49,50,52,54,55]. Further, five papers provided evidence that EM doctors experiencing psychological occupational syndromes are more likely to reduce their working hours or consider leaving their career altogether [40,41,46,49,51]. Only one paper directly considered the risk of suicide, with 10% of the sample reporting suicidal ideation to some degree [39], indicating a lack of direct examination of this vital outcome related to psychological occupational syndromes across the included literature.

Of the 12 research studies, only seven (58%) reported gender [39,42,43,46,47,49,50], and one study did so only in reference to results at the end of the second time point [40]. Ethnicity and disability were not reported in any of the included papers, and none explored whether difficulties among teams were due to issues related to discrimination or prejudice. The lack of investigation into these matters highlights critical gaps in understanding across the literature; the absence of measuring ethnicity speaks volumes, particularly considering how recently published the included papers are.

## Discussion

The primary objective of this scoping review was to establish the current state of the evidence regarding psychological occupational syndromes experienced by UK EM doctors. While only 14 papers were included in the review, the identified themes are consistent with existing research and will help inform next steps in terms of research and policy questions. With regard to our secondary objective, to identify EDI-related factors relevant to understanding psychological occupational syndromes in EM doctors, the review identified that EDI factors were neither recorded, nor considered. We also attempted to consider the impact of COVID-19. However, on reflection it may have been premature to consider this within one specific group. This review highlighted several factors purported to be relevant across psychological occupational syndromes in EM doctors, from organizational barriers to effective working practices and team functioning, personal and demographic factors, as well as the specific nature of the ED working environment itself. While more research is needed, given the commonality of themes across the different psychological occupational syndromes, and their consistency with existing research, we cautiously conclude that regardless of how distress at work is expressed, key targets for the wellbeing of the workforce have been affirmed.

Findings reflect that organizational barriers to effective working are leading to feelings of reduced autonomy and a compromised ability to work effectively within the resources available; this has resulted in a perceived reduced quality care of care and feelings of low accomplishment in EM doctors. These findings echo previous reviews examining retention among EM [8] with concerns regarding reduced quality of patient care being closely linked to the intention to leave the medical profession. Analysis of the included studies reflects that EM doctors are conflicted about remaining in their career while knowingly delivering suboptimal clinical care due to organizational barriers and/or lack of resources. This is consistent with recent research examining barriers to retention in EM [56] where compromised resources and organizational barriers influence quality of care, resulting in eroded wellbeing and further staff losses. This aligns well with the Job Demands–Resources theory of burnout [57] which recognizes the negative impact of increased work-related stress on employee wellbeing and emphasizes the need for organizational support to address this.

Notwithstanding the focus on the UK healthcare system, when compared to reviews with an international focus, the challenges are similar [8,58,59]. Whilst there is recognition that the nature of the job is emotionally demanding and high-pressure, there is an emphasis on organizational challenges such as increased workloads, staff shortages and lack of resources. However, it is likely that most studies conducted and reviewed on an international scale mostly reflect the Global North medical contexts, omitting lower-income countries where perceived difficulties may align with more basic needs [60].

With regard to our secondary objective, the lack of investigation into EDI issues in relation to the measurement or exploration of psychological occupational syndromes in UK EM doctors is deeply concerning. Without relevant data on EDI factors, we cannot account for any differences in prevalence or impact, and we cannot tailor or adjust our interventions to ensure they are accessible to all. NHS surveys highlight issues of racism and discrimination within the NHS and the negative impact this has on staff [61]. It is vital that these issues are further understood for EM doctors in the context of psychological occupational syndromes.

While the scope of this study was to seek data on those struggling with wellbeing in the workplace, it should be noted that not all doctors working in EM are reporting high levels of psychological occupational syndromes. This may relate to lack of membership to high-risk groups, such as trainee EM doctors [3]. However, it may also be associated with coping strategies and the appraisal of work demands. Research has identified that coping is associated with psychological wellbeing [62,63], and it is likely to be relevant here. Existing research indicates that individuals who work in emergency care may be more likely to have a higher tolerance for dealing with uncertainty and an increased readiness to take risks, particularly in high-pressure environments [64].

The papers in this review primarily focused on one or two syndromes, largely looking at correlated or predictive factors. This makes it difficult to decipher the exact mechanisms of development due to the variety of measures, mixed methodological quality and the lack of investigation into other relevant syndromes such as moral injury, alienation and embitterment. There is a need for higher-quality research to guide the development of operational policies to support EM doctors, for example longitudinal research that improves understanding of causal pathways. Only one study [40] collected information at several time points across 6 months, but causal mechanisms were not established. Longitudinal research demonstrates burnout in doctors as a chronic state that prevails for over 10 years [65]. This suggests that prevention is essential; intervening earlier is likely to be more effective than attempting to ‘treat’ further down the line.

As discussed, the findings from this study reflect organizational barriers and personal demographic characteristics as core factors in understanding psychological occupational syndromes in EM doctors. This suggests a necessary multi-level solution, particularly as some of these factors are likely to be contributing to the maintenance of distress in the workplace.

A recent report [66] underlines the importance of shifting focus from individual-level interventions to organizational interventions when addressing workplace wellbeing, which sits well with our findings regarding organizational barriers. NHS leaders and hospital management must work with EM doctors to consider ways to increase control and autonomy [6] which could include practical solutions such as self-rostering [45]. Many of these solutions and issues raised have already been identified in existing policy frameworks [67,68]. However, the level and success of interventions directed at ameliorating these difficulties, and whether they are embedded at a policy level in organizations, is unclear.

Certain personal and demographic characteristics such as experience, age and gender repeatedly present as risk factors and vulnerabilities, and these should be considered when planning organizational interventions. Within teams, morale needs to be prioritized [51]. This can be influenced by adherence to policies such as those outlined earlier, but more importantly, it is largely influenced by team leadership [56] and tone-setting at professional and organizational levels. EM leaders should integrate a wellbeing agenda into pre-existing departmental processes, such as debriefs and handovers [48].

On an individual level, NICE (National Institute for Health and Care Excellence) recommend evidence-based approaches such as cognitive behavioural therapy (CBT) for burnout [69]. The CBT model highlights the role of coping as key to the maintenance of a vicious cycle of job strain and burnout and identifies a target for individual-level intervention. However, this should complement organizational changes.

As reported in the COVID-19 Clinician Cohort (CoCCo) study [70] most low-level distress at work can be sufficiently ameliorated by organizational and team-level interventions, with few requiring protocolized psychological intervention. It is imperative, however, that there are services for those who need to access support, and indeed to cultivate a culture where wellbeing is a key consideration [56,70].

Although further research is needed to understand psychological occupational syndromes in EM, who they affect most and how best to treat them, we have sufficient data to confidently identify key barriers to wellbeing in the workplace. The scope of this review was restricted to psychological occupational syndromes in doctors in EM, so we have not benefitted from learning whether these identified factors associated with psychological occupational syndromes are specific to EM. However, the argument for our focus is clear; NHS emergency departments are struggling, and EM doctors are relied upon for leadership of these services. Whilst research should also focus on other professionals, roles and responsibilities vary and different strategies may be required to meet the needs of other populations.

A noted limitation of this review is the reliance on opportunity sampling and self-report methods across the included papers. Opportunity sampling limits generalizability as it may not capture the whole cohort representatively; EM doctors most impacted by psychological occupational syndromes may choose not to participate due to the additional burden on top of what they are already experiencing. Respondents may also represent EM doctors with lower levels of psychological occupational syndromes who have remained in their careers, thereby introducing potential survivorship bias. Further, it is important to note that combining a wide range of psychological conditions into one review can introduce bias in the results, such as heterogeneity bias and measurement bias. However, we note that all reviews are vulnerable to methodological biases in the included papers.

The heterogeneity of data across the included papers compromises clear interpretation of the data and the degree to which meaningful conclusions can be drawn. We are also aware that the included papers were dated from 1993 to 2024, during which there were substantial changes in NHS funding, workforce, service demand and national policy, which may have influenced the prevalence and experience of psychological occupational syndromes, reducing the ability to compare directly between studies conducted over different time points. The conclusions drawn from this review should be interpreted with this in mind.

This review has scoped the current state of the evidence and highlighted that more research is needed in the field of psychological occupational syndromes in EM. The findings of this review confirm existing research in this area, particularly that wellbeing at work is closely associated with organizational barriers, and that this is where the solution is also likely to lie. Interventions for psychological occupational syndromes must be multi-level, including system-, professional-, team- and individual-level interventions, but change must be mandated at organizational level. Improvements in wellbeing at work also rely on policy development and adherence, a step-change and national tone-setting by the government through further investment in organizational change.

## Supplementary Material

kqag080_Supplementary_Data
