# Supplementary material for: Psychological occupational syndromes in UK emergency medicine doctors: a scoping review
Source: Occup Med (Lond). 2026 Sep 28;76(7):503–14. doi: 10.1093/occmed/kqag080 (PMC13740513; doi:10.1093/occmed/kqag080)
Supplement: Supplementary file 1 [file kqag080_supplementary_data.zip › kqag080_Table 3.pdf]

Table 3 Summary of included sources (n = 17)

**Paper details**			**Extraction**						
**Author, year and area of UK**	**Timing in relation to COVID-19**	**Type of paper**	**Sample size and participants**	**Design, methods and aim of study**	**Outcomes: occupational syndrome(s) identified in paper and method of measurement**	**Type of emergency department**	**Experience of, and factors related to, the occupational syndrome(s) reported**	**Factors related to issues of Equality, Diversity and Inclusion (EDI)**	**Other relevant findings**
Burbeck et al., 2001 [39]: across the UK	Pre COVID-19	Research, Emergency Medicine Journal	350 consultants.	Quantitative cross sectional survey design investigating levels of occupational stress experienced by UK A&E consultants.	Occupational stress (psychological distress and depression). Measured using the GHQ-12 (General Health Questionnaire) and the SCL-D.	Does not specify, multicentre.	44% of participants met the threshold for psychological distress and 63% for depression.<br>Being overstretched at times, effect of working hours on personal life, lack of recognition and effect of stress on personal life were all significant predictors of higher scores of psychological distress.	Gender split of sample included.<br>Women reported significantly higher depression scores, compared to men.<br>No other EDI factors reported.	Significant positive relationship between distress scores and hours worked during the previous week.<br>Stressful incidents identified including dealing with management, relationships between colleagues and lack of beds within the hospital.
Williams et al., 1997 [40]: 27 hospitals in the South Thames Region	Pre COVID-19	Research, British Medical Journal	171 junior doctors.	Quantitative survey design with 4 time points.<br>Longitudinal questionnaire study investigating psychological distress and confidence in clinical tasks, and work-related stressors, in senior house officers across their 6 months training attachment.	Psychological distress, confidence in clinical and practical tasks.<br>Measured using a survey developed by study authors and visual analogue scales.	Does not specify, multicentre.	Lower confidence scores were related to higher psychological distress levels.<br>Communication difficulties, specific clinical presentations, organizational factors were identified as causing work related stress.	Gender split reported at one timepoint within the study.<br>At the end of the first month men's confidence levels were significantly greater than women's, but no significant gender differences were found at the end of the fourth month.<br>No other EDI factors reported.	Significant increase in confidence across 6 months training attachment.<br>Psychological distress levels did not change significantly across timepoints.

(continued)

Table 3. (continued)

Paper details			Extraction						
**Author, year and area of UK**	**Timing in relation to COVID-19**	**Type of paper**	**Sample size and participants**	**Design, methods and aim of study**	**Outcomes: occupational syndrome(s) identified in paper and method of measurement**	**Type of emergency department**	**Experience of, and factors related to, the occupational syndrome(s) reported**	**Factors related to issues of Equality, Diversity and Inclusion (EDI)**	**Other relevant findings**
Heyworth et al., 1993 [41]: across the UK	Pre COVID-19	Research, Archives of Emergency Medicine	365 junior doctors.	Quantitative cross sectional survey design, investigating work-related stress and work satisfaction in senior house officers during their A&E training.	Work related stress, work satisfaction. Measured using the Work Related Strain Inventory and Likert scales.	Does not specify, multicentre.	Participants did not report high levels of work-related stress and reported general satisfaction with their work environment. Clarity of tasks and roles was the largest predictor of job satisfaction, followed by work-related stress and team cohesiveness.	No EDI factors reported.	92.3% reported that they were unlikely to specialize in A&E.
McPherson et al., 2003 [43]: six A&E departments in North London.	Pre COVID-19	Research, Emergency Medicine Journal	37 junior doctors.	Quantitative cross sectional survey design investigating psychological distress levels in SHOs working in A&E departments.	Psychological distress measured using the GHQ-28 (General Health Questionnaire).	Does not specify, multicentre.	51% of participants scored over the threshold for psychological distress, using the General Health Questionnaire.	Gender split of sample included. No other EDI factors reported.	Coping style ‘venting’ was associated with significantly higher anxiety and depression. Coping style ‘active coping’ was associated with significantly lower anxiety and somatic complaints, and years since qualification.
Heyworth et al., 1993 [42]: across the UK	Pre COVID-19	Research, Archives of Emergency Medicine	201 consultants and senior registrars.	Quantitative cross sectional survey design investigating work-related stress in consultants and senior	Work-related stress, work satisfaction, depression. Measured using the Work-Related Strain	Does not specify, multicentre.	Results did not suggest that participants were experiencing high levels of work-related stress or depression and	Gender split of sample included. No other EDI factors reported.	The only difference between consultants and senior registrars were that consultants were more likely to

(continued)

Table 3. (continued)

**Paper details**			**Extraction**						
**Author, year and area of UK**	**Timing in relation to COVID-19**	**Type of paper**	**Sample size and participants**	**Design, methods and aim of study**	**Outcomes: occupational syndrome(s) identified in paper and method of measurement**	**Type of emergency department**	**Experience of, and factors related to, the occupational syndrome(s) reported**	**Factors related to issues of Equality, Diversity and Inclusion (EDI)**	**Other relevant findings**
				registrars working in A&E departments.	Inventory, Likert scales and the CES-D (Center for Epidemiologic Studies Depression Scale).		generally found their work satisfying. Having task and role clarity was negatively related to work stress and positively related to work satisfaction and group functioning. Work group functioning was positively related to work satisfaction.		view their work group as cohesive and smoothly functioning. Older participants reported significantly more satisfaction with their work.
Bailey et al., 2018 [44]: across the UK	Pre COVID-19	Research presented as a conference abstract, BMJ Leader	432 junior doctors.	Quantitative, prospective survey based study investigating relationships between grit, burnout, anxiety, depression and progression through training in emergency medicine (ST1-ST6).	Burnout, anxiety, depression. Measured using the Oldenburg Burnout Inventory, the GAD-7 (Generalized Anxiety Disorder questionnaire) and the PHQ-9 (Patient Health Questionnaire).	Does not specify, multicentre.	All grades of training represented high or very high-risk of burnout. Increased grit scores were significantly associated with progression through training.	No EDI factors reported.	Additional locum work was associated with significantly lower burnout scores, compared to trainees who did not do additional locum work.
Robertson, 2024 [45]: across the UK	Post COVID-19 lockdown restrictions	Research, presented as a conference abstract, Emergency Medical Journal	224 junior doctors.	Quantitative cross sectional survey design investigating the impact of self-rostering on wellbeing in emergency medicine trainees.	Need for recovery, wellbeing. Measured using the Need for Recovery Scale and Likert scales.	Does not specify, multicentre.	EM trainees who self-rostered reported significantly lower need for recovery scores, compared to trainees who were not self-rostered.	No EDI factors reported.	Trainees who were self-rostered reported feeling more in control over their work, finding their rota to interfere less with their

(continued)

Table 3. (continued)

Paper details			Extraction						
**Author, year and area of UK**	**Timing in relation to COVID-19**	**Type of paper**	**Sample size and participants**	**Design, methods and aim of study**	**Outcomes: occupational syndrome(s) identified in paper and method of measurement**	**Type of emergency department**	**Experience of, and factors related to, the occupational syndrome(s) reported**	**Factors related to issues of Equality, Diversity and Inclusion (EDI)**	**Other relevant findings**
									personal life and wellbeing and to more suitable for their training needs.
Sbaffi et al., 2020 [50]: 4 NHS trusts in North England—Leeds Teaching Hospitals, Bradford Teaching Hospitals, Airedale and North Tees and Hartlepool	Data collection pre COVID-19	Research, Journal of Medical Internet Research	101 EM doctors at any level.	Mixed methods: cross-sectional survey design with open and closed questions. Aimed to investigate causes, experience and impact of medical information overload in emergency medicine physicians.	Medical information overload and its possible impact on emergency medicine physicians. Measured using a survey developed for the study, with open and closed questions.	Does not specify, multicentre.	Large agreement that medical information overload has a serious impact on emergency medicine physicians' jobs. Information overload can lead to guideline fatigue, stress, tension, longer working hours and impaired decision-making.<br>24/7 work culture, emails, MDT communications and clinical guidelines as most concerning forms of information overload.	Gender split of sample included. No other EDI factors reported.	Younger physicians reported being more impacted by reduced social activities due to experiencing information overload, and increased impaired decision-making and inaccurate clinical judgements, compared to older participants. Physicians are concerned about impact on patient care and wellbeing because of the effects of information overload.
Dasan et al., 2014 [49]: across the UK	Pre COVID-19	Research, Emergency Medicine Journal	681 consultants.	Mixed methods study investigating compassion satisfaction and	Compassion fatigue (burnout and secondary traumatic stress)	ED = 524 (79%) Major Trauma Centre = 132 (19.9%)	The majority of participants reported scores in the average range for compassion	Gender split of sample included. No other	Compassion satisfaction appeared to reduce over the

(continued)

Table 3. (continued)

**Paper details**			**Extraction**						
**Author, year and area of UK**	**Timing in relation to COVID-19**	**Type of paper**	**Sample size and participants**	**Design, methods and aim of study**	**Outcomes: occupational syndrome(s) identified in paper and method of measurement**	**Type of emergency department**	**Experience of, and factors related to, the occupational syndrome(s) reported**	**Factors related to issues of Equality, Diversity and Inclusion (EDI)**	**Other relevant findings**
				compassion fatigue in UK consultants in emergency medicine, using questionnaire and follow up interviews.	and compassion satisfaction (feeling of pleasure from helping others). Measured using the ProQOL (Professional Quality of Life Questionnaire). Follow up interviews with 12 consultants.	Minor injuries = 1 (0.2%) Missing data = 6 (0.9%)	satisfaction and burnout and in the low range for secondary traumatic stress. 15/681 reported compassion fatigue, two of these had high burnout scores and 1 had a high secondary traumatic stress score. Years worked as a consultant and working in a designated trauma centre were key predictors of compassion satisfaction. Marital status and years worked as a consultant were key predictors of burnout and secondary traumatic stress. Perceived lack of control, repetitiveness, unreasonable demands were all factors relevant to experiencing feelings of compassion fatigue.	EDI factors reported.	first 10 years of working as a consultant and then increase after 20 years. The same finding was reported for burnout and secondary traumatic stress.
Fitzgerald et al., 2016 [46]: Southwest England	Pre COVID-19	Research, Emergency Medicine Journal	18 consultants.	Qualitative interview based research exploring psychological distress and wellbeing in	Psychological distress and wellbeing. Measured using semi structured interview.	Type 1 EDs (n = 5; consultant led 24 hour A&E).	Key themes identified were: 'systemic pressures', 'physical and mental strain' and 'managing the challenges'.	Gender split of sample included. No other EDI factors reported.	Social support at work, role development and supporting problem solving within the emergency

(continued)

Table 3. (continued)

Paper details			Extraction						
**Author, year and area of UK**	**Timing in relation to COVID-19**	**Type of paper**	**Sample size and participants**	**Design, methods and aim of study**	**Outcomes: occupational syndrome(s) identified in paper and method of measurement**	**Type of emergency department**	**Experience of, and factors related to, the occupational syndrome(s) reported**	**Factors related to issues of Equality, Diversity and Inclusion (EDI)**	**Other relevant findings**
				emergency medicine consultants.			Unsustainable demands and workload, diminished sense of achievement, uncontrollable patient flow, reduced resources, physical and mental exhaustion, threat to professional identity, uncertainty around personal sustainability, difficulty balancing work and life, were all factors reported as having an impact on emergency medicine consultants' wellbeing and patient care.		department at an organizational level were identified as ways that individuals could feel their career as an EM consultant was more sustainable.
Howard et al., 2018 [47]: Manchester	Pre COVID-19	Research, Emergency Medicine Journal	17 EM doctors of any level.	Qualitative interview based research to understand impact of clinical events on the wellbeing of emergency medicine physicians and which ones cause emotional disruption.	Emotional disruption caused by clinical events in the emergency department. Measured using narrative style interviews.	Does not specify.	Key clinical events identified were young or traumatic deaths, events that related to their own lives, witnessing the consequences of death on relatives, burden of responsibility and conflict within the workplace. Emotional distress, reduced self-confidence, withdrawal and active avoidance of emotions were all symptoms reported due to these clinical events.	Gender split of sample included. No other EDI factors reported.	Participants also reported that becoming withdrawn and non-medical friends or family not knowing how best to support them both had impacts on their personal relationships.

(continued)

Table 3. (continued)

Paper details			Extraction						
**Author, year and area of UK**	**Timing in relation to COVID-19**	**Type of paper**	**Sample size and participants**	**Design, methods and aim of study**	**Outcomes: occupational syndrome(s) identified in paper and method of measurement**	**Type of emergency department**	**Experience of, and factors related to, the occupational syndrome(s) reported**	**Factors related to issues of Equality, Diversity and Inclusion (EDI)**	**Other relevant findings**
Park et al., 2019 [48]: Edinburgh	Pre COVID-19	Research, presented as a conference abstract, Emergency Medicine Journal	10 junior doctors.	Qualitative interview based research exploring the perceptions of handover in the ED and whether it is stressful, in junior doctors.	Stress relating to ED handovers. Measured using interviews.	Does not specify.	Four themes were identified: “stress decreases as familiarity increases”, “time pressure is an ongoing stressor”, “handover as solace” and “It’s nice to be nice”. Junior doctors reported that wanting to make a good impression, time pressure organizational hierarchy and unfamiliarity as factors that make handovers stressful.	No EDI factors reported.	Handovers that allow for morale boosting, education and socialization can help reduce stress experienced by junior doctors.
Ghodrat, 2018 [51]: Merseyside	Pre COVID-19	Opinion piece, RCEM: gray literature	n/a	Expert opinion: RCEM blog post discussing author’s own experience of burnout.	Discussed burnout.	Does not specify.	Organizational factors contributing to burnout reported as low team morale, full corridors, serious understaffing and underfunding. Personal factors were noted to be reduced pleasure from activities that used to bring enjoyment. Physical symptoms were chronic exhaustion, mental slowing, impaired concentration. The author reported that their experience	No EDI factors reported.	

(continued)

Table 3. (continued)

Paper details			Extraction						
**Author, year and area of UK**	**Timing in relation to COVID-19**	**Type of paper**	**Sample size and participants**	**Design, methods and aim of study**	**Outcomes: occupational syndrome(s) identified in paper and method of measurement**	**Type of emergency department**	**Experience of, and factors related to, the occupational syndrome(s) reported**	**Factors related to issues of Equality, Diversity and Inclusion (EDI)**	**Other relevant findings**
							of being exhausted and burnt out resulted in them ignoring symptoms of a stroke, calling for changes in the system and work environment to reduce the risk of burnout and its possible severe consequences.		
Henderson, 2022 [52]: Across the UK	During COVID-19	Opinion commentary, RCEM: gray literature	n/a	Expert opinion: Commentary regarding the findings related to EM doctors in the GMC training survey	Discussed burnout.	Not applicable.	High risk of burnout for EM trainees, as well as stress and exhaustion, which is leading to staff considering reducing working hours or taking a break from their career. Burnout among EM trainees is impacting patient care and their own learning. Current working conditions in the EM are unsustainable.	No EDI factors reported.	
Jones, 2012 [53]: Manchester	Pre COVID-19	Opinion piece, St Emlyn's Blog: gray literature	n/a	Expert opinion: Opinion piece discussing own experience of burnout and coping	Discussed burnout.	Does not specify.	Experience of insomnia and overthinking about work. Recognition that certain coping strategies are more adaptive, such as problem-focused coping strategies and acceptance	No EDI factors reported.	

(continued)

Table 3. (continued)

Paper details			Extraction						
**Author, year and area of UK**	**Timing in relation to COVID-19**	**Type of paper**	**Sample size and participants**	**Design, methods and aim of study**	**Outcomes: occupational syndrome(s) identified in paper and method of measurement**	**Type of emergency department**	**Experience of, and factors related to, the occupational syndrome(s) reported**	**Factors related to issues of Equality, Diversity and Inclusion (EDI)**	**Other relevant findings**
							and adaptive emotion-focused strategies, as opposed to denial, disengagement, substance use and self-blame. The author reflected that they weren't clear how they had developed these more effective strategies over time and were concerned how they could ensure junior doctors can develop these too.		
Heyworth, 2004 [54]: Southampton	Pre COVID-19	Opinion piece, Emergency Medicine Australasia	n/a	Expert opinion: Commentary on stress in the emergency department.	Discussed stress in the emergency department	Not applicable.	The factors that make emergency medicine rewarding can also cause stress, which can make it a difficult balancing act. The nature of the emergency medicine work environment may mean that subtle signs of stress in colleagues are easily missed, such as lower mood, withdrawal and irritability. There is a need to target stressors that can be changed such as workload and develop coping strategies for	No EDI factors reported.	

(continued)

Table 3. (continued)

Paper details			Extraction						
**Author, year and area of UK**	**Timing in relation to COVID-19**	**Type of paper**	**Sample size and participants**	**Design, methods and aim of study**	**Outcomes: occupational syndrome(s) identified in paper and method of measurement**	**Type of emergency department**	**Experience of, and factors related to, the occupational syndrome(s) reported**	**Factors related to issues of Equality, Diversity and Inclusion (EDI)**	**Other relevant findings**
							those that are part of the nature of emergency medicine such as witnessing pain and unexpected deaths.		
Hassan, 2015 [55]: Leeds	Pre COVID-19	Opinion piece, Emergency Medicine Journal	n/a	Expert opinion: Commentary on Dasan et al.'s [49] paper.	Discussed compassion fatigue, burnout.	Not applicable.	Workload, inflexible organizations, work-life imbalances, loss of autonomy have all been related to emotional exhaustion. Experience of work related stress can lead to reduced satisfaction, and poor career retention in emergency medicine. Support is essential in reducing the impact of work related stressors experienced by emergency medicine doctors.	No EDI factors reported.	
