# Supplementary material for: Psychological occupational syndromes in UK emergency medicine doctors: a scoping review
Source: Occup Med (Lond). 2026 Sep 28;76(7):503–14. doi: 10.1093/occmed/kqag080 (PMC13740513; doi:10.1093/occmed/kqag080)
Supplement: Supplementary file 1 [file kqag080_supplementary_data.zip › 25-OP-182 Supplementary table FINAL.doc]

**Scoping Review Search Strategies**

**Embase**

'physician'/exp OR ‘physician$’:ti,ab OR 'doctor'/exp OR ‘doctor$’:ti,ab OR ‘trainee$’:ti,ab OR ‘consultant$’:ti,ab OR ‘emergency physician’/exp OR ‘emergency physician$’:ti,ab

AND

Burnout/exp OR ‘burnout’:ti,ab OR ‘professional burnout’/exp OR ‘professional burnout’:ti,ab OR depersonalization/exp OR ‘depersonalization’:ti,ab OR depersonalisation/exp OR ‘depersonalisation’:ti,ab OR ‘compassion fatigue’/exp OR ‘compassion fatigue’:ti,ab OR ‘occupational stress’:ti,ab OR ‘job stress’/exp OR ‘job stress’:ti,ab OR ‘emotional exhaustion’/exp OR ‘emotional exhaustion’:ti,ab OR ‘cynic*’:ti,ab OR ‘reduced personal accomplishment’:ti,ab OR ‘embitterment’:ti,ab OR ‘alienation’/exp OR ‘alienation’:ti,ab OR ‘moral injur*’:ti,ab OR ‘empathy fatigue’:ti,ab OR ‘estrangement’:ti,ab OR ‘professional efficacy’:ti,ab OR ‘job burnout’:ti,ab OR ‘work stress’:ti,ab OR ‘empathy distress’:ti,ab OR ‘moral distress’:ti,ab

AND

‘emergency medicine’/exp OR ‘emergency medic*’:ti,ab OR ‘emergency health service’/exp OR ‘emergency health service$’:ti,ab OR ‘emergency medical service$’:ti,ab OR ‘emergency service$’:ti,ab OR ‘hospital emergency service’/exp OR ‘hospital emergency service$’:ti,ab OR ‘trauma cent*’:ti,ab OR ‘emergency treatment’/exp OR ‘emergency treatment$’:ti,ab OR ‘emergency ward’/exp OR ‘emergency ward$’:ti,ab OR ‘emergency department$’:ti,ab OR ‘emergency care’/exp OR ‘emergency care’:ti,ab OR ‘accident and emergency’:ti,ab OR ‘A&E’:ti,ab OR ‘emergency room$’:ti,ab

Filters:

- Published in English language

**APA Psycnet (including PsycExtra) for grey literature)**

Doctor*[title] OR doctor*[abstract] OR physician*[title] OR physician*[abstract] OR physicians[index terms] OR registrar*[title] OR registrar*[abstract] OR trainee*[title] OR trainee*[abstract] OR consultant*[title] OR consultant*[abstract]

AND

Burnout[title] OR burnout[abstract] OR burnout[index terms] OR “job burnout”[title] OR “job burnout”[abstract] OR “job burnout”[index terms] OR depersonalization[title] OR depersonalization[abstract] OR depersonalization[index terms] OR depersonalisation[title] OR depersonalisation[abstract] OR “compassion fatigue”[title] OR “compassion fatigue”[abstract] OR “compassion fatigue”[index terms] OR “occupational stress”[title] OR “occupational stress”[abstract] OR “occupational stress”[index terms] OR “job stress”[title] OR “job stress”[abstract] OR “work stress”[title] OR “work stress”[abstract] OR “emotional exhaustion”[title] OR “emotional exhaustion”[abstract] OR “emotional exhaustion”[index terms] OR cynic*[title] OR cynic*[abstract] OR cynicism[index terms] OR “reduced personal accomplishment”[title] OR “reduced personal accomplishment”[abstract] OR “professional efficacy”[title] OR “professional efficacy”[abstract] OR embitterment[title] OR embitterment[abstract] OR alienation[title] OR alienation[abstract] OR alienation[index terms] OR “moral injur*”[title] OR “moral injur*”[abstract] OR “moral injury”[index terms] OR “empathy fatigue”[title] OR “empathy fatigue”[abstract] OR estrangement[title] OR estrangement [abstract] OR “empathy distress”[title] OR “empathy distress” [abstract] OR “moral distress” [title] OR “moral distress”[abstract]

AND

“emergency medicine”[index terms] OR “emergency medic*”[title] OR “emergency medic*”[abstract] OR “emergency services”[index terms] OR “emergency service*”[title] OR “emergency service*”[abstract] OR “trauma cent*”[title] OR “trauma cent*[abstract] OR “emergency treatment*”[title] OR “emergency treatment*”[abstract] OR “emergency medical service*”[title] OR “emergency medical service*”[abstract] OR “emergency department*”[title] OR “emergency department*”[abstract] OR “emergency care”[title] OR “emergency care”[abstract] OR “accident and emergency”[title] OR “accident and emergency”[abstract] OR “A&E”[title] OR “A&E”[abstract] OR “emergency room*”[title] OR “emergency room*”[abstract] OR “emergency ward*” [title] OR “emergency ward*”[abstract] OR “emergency health service*”[title] OR “emergency health service*” [abstract] OR “hospital emergency service*”[title] OR “hospital emergency service*”[abstract]

AND

english [language]

**CINAHL**

TI doctor OR AB doctor OR TI physician OR AB physician OR (MH “Physicians+”) OR TI registrar OR AB registrar OR TI trainee OR AB trainee OR TI consultant OR AB consultant

AND

(MH “Burnout, Professional+”) OR TI burnout OR AB burnout OR TI “job burnout” OR AB “job burnout” OR (MH “Depersonalization”) OR TI depersonali?ation OR AB depersonali?ation OR (MH “Compassion Fatigue”) OR TI “compassion fatigue” OR AB “compassion fatigue” OR (MH “Stress, Occupational+”) OR TI “occupational stress” OR AB “occupational stress” OR TI “job stress” OR AB “job stress” OR TI “work stress” OR AB “work stress” OR (MH “Emotional Exhaustion”) OR TI “emotional exhaustion” OR AB “emotional exhaustion” OR TI cynic* OR AB cynic* OR TI “reduced personal accomplishment” OR AB “reduced personal accomplishment” OR TI “professional efficacy” OR AB “professional efficacy” OR TI embitterment OR AB embitterment OR TI alienation OR AB alienation OR TI “moral injur*” OR AB “moral injur*” OR TI “empathy fatigue” OR AB “empathy fatigue” OR TI estrangement OR AB estrangement OR TI “empathy distress” OR AB “empathy distress” OR TI “moral distress” OR AB “moral distress”

AND

(MH “Emergency Medicine”) OR TI “emergency medic*” OR AB “emergency medic*” (MH “Emergency Service+”) OR TI “emergency service*” OR AB “emergency service*” OR (MH “Emergency Medical Services+”) OR TI “emergency medical service*” OR AB “emergency medical service*” OR (MH “trauma centers”) OR TI “trauma cent*” OR AB “trauma cent*” OR (MH “Emergency Treatment+”) OR TI “emergency treatment*” OR AB “emergency treatment*” OR TI “emergency department*” OR AB “emergency department*” OR (MH “Emergency Care+”) OR TI “emergency care” OR AB “emergency care” OR TI “accident and emergency” OR AB “accident and emergency” OR TI “A&E” OR AB “A&E” OR TI “emergency room*” OR AB “emergency room*” OR TI “emergency ward*” OR AB “emergency ward*” OR TI “emergency health service*” OR AB “emergency health service*” OR TI “hospital emergency service*” OR AB “hospital emergency service*”

Filters:

- Published in English language

**Appendix C**

**Scoping Review Data Extraction Form**

| **Scoping Review Details** |  |
| --- | --- |
| Scoping Review title: | What do we know about the occupational syndromes experienced by NHS doctors working in emergency medicine? A scoping review. |
| Reviewer: |  |
| Date: |  |
| Inclusion criteria:   - UK doctors who work in emergency medicine. - ‘Occupational syndromes’ e.g. burnout, compassion fatigue, alienation, embitterment, moral injury. - Any evidence source other than review | Does it meet criteria? |
| **Evidence source details and characteristics** |  |
| Citation details (author/s, date, title, journal, volume, issue, pages) |  |
| Literature type (primary research, conference presentation, opinion piece, gray literature) |  |
| Research questions/aims |  |
| County/area of the UK |  |
| Type of emergency department (e.g. Type 1 ED, Type 2 ED, Type 3 ED, major trauma centre, etc) |  |
| Pre COVID-19, during COVID-19 lockdown restrictions, or post COVID-19 lockdown restrictions |  |
| Sample size |  |
| Recruitment method |  |
| Age range of sample |  |
| Gender |  |
| Ethnicity |  |
| Years in profession |  |
| Seniority |  |
| Leadership role or responsibility? |  |
| Hours worked per week/part time or full time |  |
| **Details/Results extracted from source of evidence** |  |
| Methodology |  |
| Measures used |  |
| Data collection method used |  |
| Outcomes and definition (how the paper has defined the outcome they have used e.g. burnout, compassion fatigue) |  |
| Data analysis/analytical approach |  |
| Key findings relevant to the review including data such as participant quotes in qualitative studies |  |
| Any findings specific to equality, diversity and inclusion factors |  |
| Other factors that might be relevant to working conditions e.g. mentions overstretched teams |  |
| Key conclusions by study authors  (approx. 3 brief bullet points) |  |
| Other information relevant to the review questions |  |
